# Evaluating the Real-World Pharmacokinetics of Risperidone ISM^®^ in Routine Clinical Practice

**DOI:** 10.3390/biomedicines13020384

**Published:** 2025-02-06

**Authors:** Francisco José Toja-Camba, María Vidal-Millares, María José Duran-Maseda, Manuel Arrojo-Romero, María Puente-Iglesias, Gonzalo Hermelo-Vidal, Carolina Feitosa-Medeiros, Anxo Fernández-Ferreiro, Cristina Mondelo-García

**Affiliations:** 1 Pharmacy Department, University Clinical Hospital of Santiago de Compostela (SERGAS), 15706 Santiago de Compostela, Spain; kikotoja@gmail.com (F.J.T.-C.); maria.puente.iglesias@sergas.es (M.P.-I.); 2 FarmaCHUSLab Group, Health Research Institute of Santiago de Compostela (IDIS), 15706 Santiago de Compostela, Spain; zalohermelo@gmail.com (G.H.-V.); carolinafeimed@gmail.com (C.F.-M.); 3 Psychiatry Department, University Clinical Hospital of Santiago de Compostela (SERGAS), 15706 Santiago de Compostela, Spain; maria.vidal.millares@sergas.es (M.V.-M.); maria.jose.duran.maseda@sergas.es (M.J.D.-M.); manuel.arrojo.romero@sergas.es (M.A.-R.)

**Keywords:** risperidone, ISM, pharmacokinetics, schizophrenia

## Abstract

**Background/Objectives**: Risperidone ISM^®^ is a long-acting injectable (LAI) formulation approved for monthly administration in schizophrenia treatment. It employs innovative in situ microimplant technology for biphasic drug release, achieving immediate and sustained therapeutic plasma concentrations without the need for oral supplementation or loading doses. This study evaluates the pharmacokinetic profile of Risperidone ISM^®^ in a real-world clinical setting, focusing on plasma concentrations of the active moiety (Risperidone + 9-OH-Risperidone). **Methods**: An observational study was conducted to measure plasma concentrations of patients receiving Risperidone ISM^®^ (75 mg or 100 mg). Samples were collected at pre-dose, 2 h, 24 h, 14 days, 21 days, and 25 days post-injection. Pharmacokinetic parameters, including Cmax, Cmin, Tmax, and AUC, were calculated and stratified by dose (75 mg and 100 mg) and injection site (gluteal vs. deltoid). **Results**: A total of 44 patients were included. Therapeutic plasma levels were reached within hours post-injection and sustained throughout the 28-day interval. Cmin values averaged 34.4 ng/mL and 36.1 ng/mL for the 75 mg and 100 mg doses, respectively. The median Tmax occurred at 24 h with a mean Cmax of 55.7 ng/mL and 59 ng/mL for 75 mg and 100 mg, respectively. Higher systemic exposure was observed for deltoid administration. Significant interindividual variability was noted, with 45.4% of patients exhibiting trough levels outside the therapeutic range. **Conclusions**: Risperidone ISM^®^ achieves rapid and sustained therapeutic plasma levels, offering significant benefits in schizophrenia treatment. However, the high interindividual variability observed must be thoroughly studied, and the contributing factors identified to ensure the therapy is as effective and safe as possible.

## 1. Introduction

Schizophrenia is a chronic condition that manifests through both positive and negative symptoms, affecting cognition and emotional well-being. It is estimated that approximately 0.7–1% of the world population suffers from this condition, currently affecting 24–25 million people worldwide, according to data from the World Health Organization [1,2].

The treatment for schizophrenia primarily relies on antipsychotic medications, but their effectiveness often varies greatly between individuals and even within the same individual over time. As a result, their outcomes frequently fall short of expectations [3]. Based on the law of mass action, which implies that pharmacologic effects are concentration-dependent in relation to both therapeutic improvement and adverse drug reactions, Therapeutic Drug Monitoring (TDM) assumes that there is a specific drug concentration range in the blood for maximal effectiveness and acceptable safety, known as the therapeutic reference range. Specifically for Risperidone, relationships between drug concentrations in the blood and clinical effectiveness have been reported [4,5].

One of the biggest challenges with oral antipsychotic formulations is ensuring adherence. Various factors contribute to non-adherence, including missed doses, side effects experienced during treatment, and poor insight into the condition, all of which influence medication intake. Studies that define adherence as taking medication at least 75% of the time indicate that approximately 50% of treated patients fail to adhere to their prescribed regimens [6,7]. This issue has been associated with the risk of relapse and disease progression, and it has been partially addressed with the introduction of typical long-acting injectable (LAI) antipsychotics [8]. The subsequent development of atypical LAI antipsychotics further improved the safety profile of these treatments, which are administered at extended intervals ranging from two weeks to six months [9,10]. Despite the advantages of LAIs, several barriers limit their widespread use, including challenges in defining the target patient population, uncertainty about the optimal timing for initiating LAI treatment, and the need for education and training for healthcare providers [11].

Specifically, Risperidone, a second-generation antipsychotic, has shown efficacy in managing both psychotic and mood-related symptoms [12]. The first intramuscular LAI available for this drug allowed patients with schizophrenia stabilized on oral antipsychotics to receive a single dose every two weeks (Risperdal Consta, Janssen-Cilag, Raritan, NJ, USA) [13]. More recently, in February 2022, Risperidone ISM**^®^** (Okedi, Rovi, Madrid, Spain) received valid marketing authorization throughout the European Union for adult patients who have demonstrated tolerability and effectiveness with oral Risperidone [14]. It allows a single administration every 28 days, which is twice the interval permitted by the previously available LAI formulation. This is made possible by ISM**^®^** technology, a stable solid polymer matrix system composed of L-lactide and glycolide monomers whose biodegradation kinetics provide both rapid and sustained drug release over 28 days [15]. In this sense, the acronym ISM should not be confused with what has been known in the jargon of pharmaceutical technology as “in situ microparticles”, as pointed out by Anta and Mata in a letter to the editor following the review published by Clark and Taylor on new formulations of Risperidone [16,17]. The technology refers to “in situ microimplants”, as can be seen in the registered patent [18]. Despite this, other recent works continue to refer to the acronym as microparticles [19].

Regarding Risperidone systemic metabolism, it primarily undergoes hepatic biotransformation mediated by CYP2D6, resulting in its main active metabolite, 9-OH-Risperidone [20]. Concerning pharmacokinetics, the German Working Group for Neuropsychopharmacology and Pharmacopsychiatry (AGNP) reflected in their AGNP Consensus Guidelines for Therapeutic Drug Monitoring that the therapeutic reference range for the combined concentrations of Risperidone and its active metabolite 9-OH-Risperidone (active moiety), in terms of trough or minimum concentration, is 20–60 ng/mL [5].

Risperidone ISM**^®^** contains the drug in a suspension delivery system that features a biphasic release process. According to its summary of product characteristics (SmPCs), following intramuscular injection, a small amount of the medication is immediately released at the time of injection, providing instant plasma concentrations. After an initial peak concentration, the mean plasma levels steadily decline until day 14, after which they rise again to reach a second peak concentration approximately between days 21 and 24. Following the second peak, plasma concentrations gradually decrease over time. The suspension has a prolonged effect, delivering sustained therapeutic plasma concentrations that are maintained throughout the 28-day dosing interval [21]. Consequently, the main advantage of this formulation lies in achieving therapeutic plasma concentrations of Risperidone immediately within the first few hours after administration and maintaining them throughout the dosing period without the need for oral supplementation or loading doses [22,23].

In cases of treatment resistance, dose adjustments rely on a trial-and-error approach due to the current inability to predict therapeutic outcomes. This, combined with the frequent occurrence of severe adverse effects, often leads patients to discontinue antipsychotic therapies. In this sense, personalized medicine is an essential tool for applying population stratification criteria, enabling the optimization of current and future therapeutic strategies. Specifically, its application in antipsychotic therapy represents a turning point in the therapeutic management of patients, as it provides deeper insights into the relationship between drug exposure and clinical response, both in terms of efficacy and safety [24].

In this context, this work aims to evaluate the plasma concentrations of the active moiety (Risperidone + 9-OH-Risperidone) in a cohort of patients treated with Risperidone ISM^®^ to elucidate how this new formulation behaves in a real clinical setting.

## 2. Materials and Methods

An observational study of a cohort of patients under Risperidone ISM**^®^** (Okedi, Rovi) treatment was conducted to evaluate the plasma concentrations. The authors declare that all procedures performed were in accordance with the ethical standards of the relevant national and institutional committees on human experimentation and with the 1975 Declaration of Helsinki (as revised in 2008). All procedures involving patients were approved by the Drug Research Ethics Committee of Galicia (2021/285), and written informed consent was obtained from all subjects. This study included patients who received at least four doses of Risperidone ISM**^®^**. Exclusion criteria were age < 18 years, pregnancy, and cognitive impairment. Three blood samples were obtained from each patient at pre-dose, 2 h, and 24 h post-injection. In addition, in a subgroup of 23 patients, three more extractions were performed at 14, 21, and 25 days in order to characterize the second release phase characteristic of this formulation. Demographic characteristics and biochemical data, including gender, age, height, weight, and liver and kidney function, were recorded. Also, the Risperidone ISM**^®^** site of injection and dose were collected.

Samples were stored at −20 °C until analysis. The analysis was performed with Alinity C, a photometric assay for the active moiety of Risperidone, using the MyCare Psychiatry Kit developed, validated, and tested for cross-reactions by Saladax Biomedical (Bethlehem, PA, USA) for total Risperidone in whole blood. The measurement was taken at a wavelength of 604 nm. The lower limit of quantification of the kit is 16 ng/mL, and the upper limit of quantification is 120 ng/mL. Samples exceeding the upper limit of quantification were diluted for analysis.

Descriptive statistics, including geometric mean, coefficient of variation (CV%), arithmetic mean and standard deviation (SD), median and interquartile range (IQ), and range (minimum and maximum) were calculated.

## 3. Results

### 3.1. Study Population

A total of 44 patients under Risperidone ISM**^®^** (Okedi, Rovi) treatment were recruited. The median age was 51 years (IQ: 41–62), with 63.6% being male participants. The majority of injections were administered in the gluteus (70.4%), while the remaining 29.5% were performed in the deltoid. Baseline and demographic characteristics are available in Table 1.

### 3.2. Plasma Concentrations After Initial Drug Release

**Figure 1:**
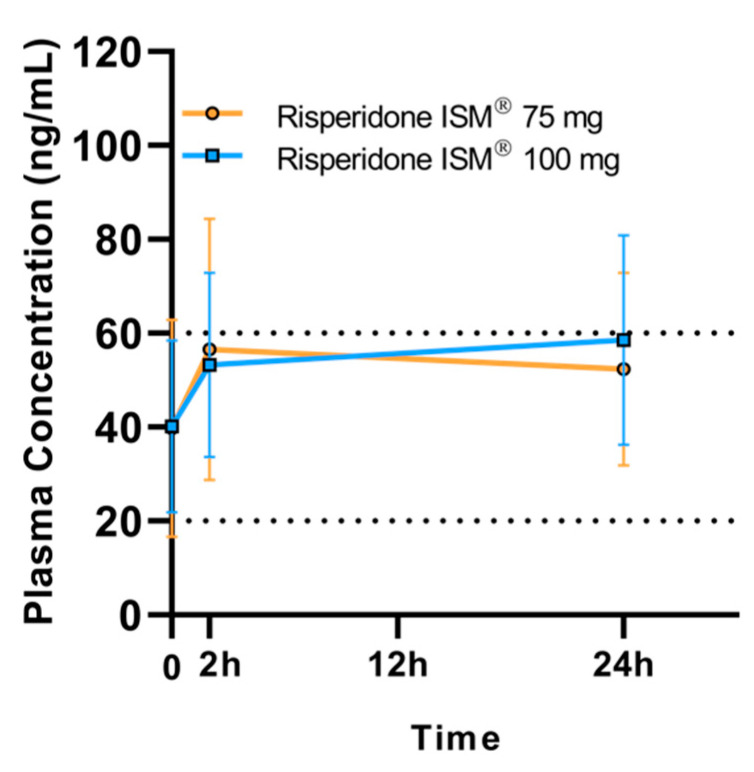
Arithmetic mean (SD) plasma concentrations of the active moiety at pre-dose (trough), 2 h, and 24 h post-dose. Black dashed lines represent the Risperidone therapeutic reference range.

Plasma concentrations measured pre-dose, 2 h, and 24 h post-dose revealed rapid initial absorption. At 2 h, concentrations reached values above the lower limit of the proposed therapeutic range. At 24 h, both doses reached high values; although the results are reported as mean values, it is important to highlight that certain patients clearly surpassed this range (Figure 1).

**Figure 2:**
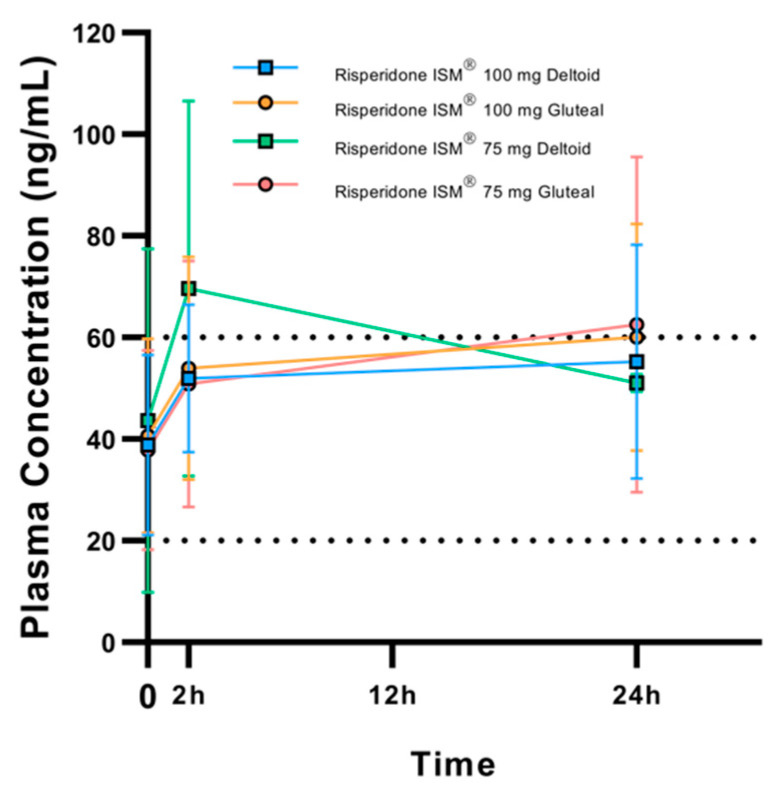
Arithmetic mean (SD) plasma concentrations of active moiety at pre-dose (trough), 2 h, and 24 h post-dose, taking into account the injection site. Black dashed lines represent the Risperidone therapeutic reference range.

When stratifying by injection site, it is observed that the elevated plasma concentrations at 24 h in the groups receiving 75 mg in the gluteus (62.5 ± 33 ng/mL) and 100 mg in the gluteus (60.05 ± 22.3 ng/mL) exceeded 60 ng/mL (Figure 2). As can be seen in both Figure 1 and Figure 2, there is a high interindividual variability in plasma concentrations of active moiety.

### 3.3. Plasma Concentrations Throughout the Dosing Interval

**Figure 3:**
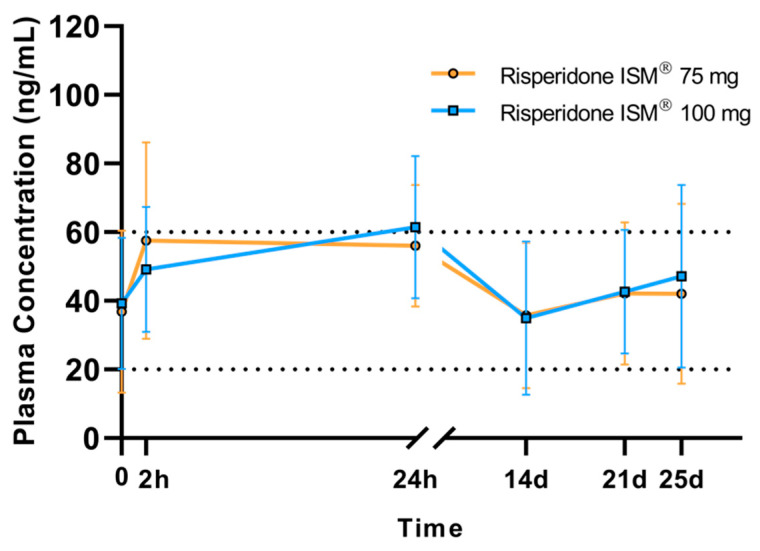
Arithmetic mean (SD) plasma concentrations of the active moiety at pre-dose (trough), 2 h, 24 h, 14 days, 21 days, and 25 days post-dose. Black dashed lines represent the Risperidone therapeutic reference range.

In a subgroup of 23 patients, samples were extracted throughout the dosage interval. The minimum concentration (Cmin) reached for patients treated with a 75 mg dose was 34.4 ng/mL, and for patients treated with a 100 mg dose, it was 36.1 ng/mL (geometric mean). Due to the high variability observed, it was analyzed how many patients were not within the reference therapeutic range at time 0 (Ctrough); 45.4% of the patients were outside the range. Specifically, 34% of those treated with 100 mg (below: 15.9%; above: 18.1%) and 11.3% of those treated with 7 mg (below: 6.8%; above: 4.5%). The maximum concentration was reached at 24 h (Tmax) for 100 mg and 2 h for 75 mg, with values of 55.7 in the case of 75 mg and 59 in the case of 100 mg. At 14 days, both patients treated with 75 mg and 100 mg showed a similar decrease in mean concentrations, which remained within the therapeutic range despite high variability in which it can be observed that there were patients whose concentrations decreased below 20 ng/mL (Figure 3.) From this point on, plasma concentrations increased again, reaching their maximum at 21–25 days.

**Figure 4:**
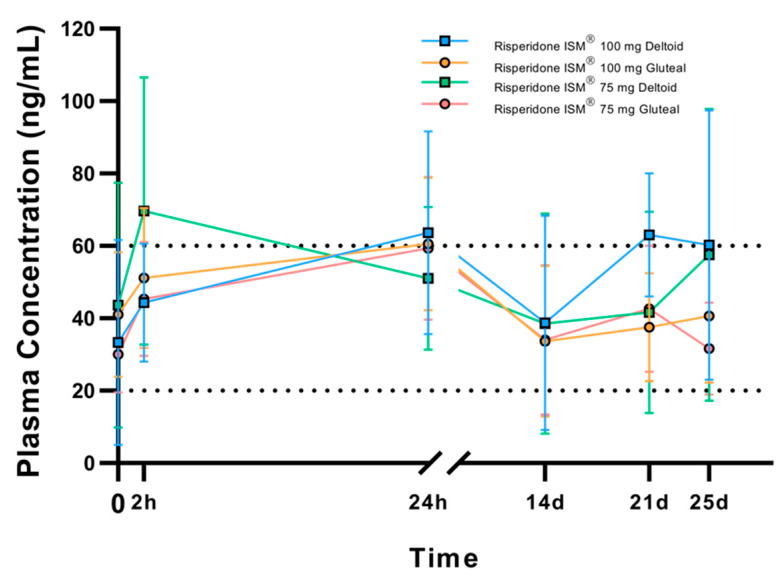
Arithmetic mean (SD) plasma concentrations of the active moiety at pre-dose (trough), 2 h, 24 h, 14 days, 21 days, and 25 days post-dose considering the injection site. Black dashed lines represent the Risperidone therapeutic reference range.

When stratifying patients by dose and injection site, we observed a behavior similar to the previous one. However, concentrations at 21 and 25 days appear higher for patients who received the drug in the deltoid (Figure 4). Again, in this subgroup of 23 patients, we observed a high variability of plasma concentrations with coefficients of variation of 45.7% for Cmax and 40.9% for AUC in the 100 mg deltoid group (Table 2).

## 4. Discussion

Risperidone ISM**^®^** is a technology that enables the formation of an in situ solid and stable polymeric matrix system containing Risperidone. After reconstitution and intramuscular injection, a small amount of the medication is immediately released at the time of administration, providing instant plasma concentrations. In addition, it precipitates in situ to form a matrix by solvent diffusion to body fluids, which biodegrades slowly, providing a sustained and controlled drug release for up to 28 days [16].

The FDA approved RBP-7000 in 2018, making it the first long-acting Risperidone formulation for monthly administration that achieved therapeutic concentrations in the initial doses. However, it is currently unavailable [25]. Years later, the SmPC for aripiprazole monthly (Abilify Maintena**^®^**, Otsuka, Tokyo, Japan) included a new dosing regimen for initiating injectable therapy, which required not only a loading dose but also oral aripiprazole supplementation to maintain therapeutic concentrations during the initiation of treatment [26].Therefore, the approval of Risperidone ISM**^®^** in 2022 marks the emergence of the only monthly LAI Risperidone currently available that achieved therapeutic concentrations within hours of administration without the need for oral treatment supplementation. In this regard, other monthly LAI atypical antipsychotics require a loading dose to attain steady-state concentrations when switching from oral treatment to their injectable formulations because they do not have a rapid optimal release [13,27].

This study aims to assess the plasma concentrations of the active moiety (Risperidone + 9-OH-Risperidone) in a cohort of patients receiving treatment with Risperidone ISM**^®^** in a real clinical setting. In this regard, several aspects must be highlighted and discussed with the available literature.

With regard to Risperidone metabolism, it is processed in the liver through biotransformation mediated by CYP2D6, leading to the formation of its primary active metabolite, 9-OH-Risperidone [20]. However, as stated by Vermeulen et al., extensive CYP2D6 metabolizers have lower Risperidone concentrations and higher 9-OH-Risperidone concentrations compared to poor metabolizers. As a result, the combined pharmacokinetics of Risperidone and 9-OH-Risperidone, after single or multiple doses, are similar in extensive and poor metabolizers and, therefore, independent of the metabolic status of the patient [28]. Based on this, pharmacogenetic analysis was not performed in the present study.

Concerning Risperidone pharmacokinetics, the AGNP reflected in their Consensus Guidelines for Therapeutic Drug Monitoring that the therapeutic range for the combined concentrations of Risperidone and its active metabolite 9-OH-Risperidone (active moiety) is 20–60 ng/mL [5]. According to the SmPC of Risperidone ISM, the mean Cmin plasma concentrations of the active moiety after repeated administration of intramuscular injections of 75 mg and 100 mg are 17.6 (below the recommended therapeutic range) and 28.9, respectively.

Previously published studies report a steady-state Cmin for a dose of 75 mg administered in the gluteal and deltoid of 12.6 and 9.5 ng/mL, respectively. Another more recent study, also performed at steady state, obtains a Cmin value of 21.2 for the 100 mg dose administered in the gluteus [22,29]. In our case, the Cmin obtained in the gluteus for the 75 mg dose is 33.6 ng/mL, which is considerably higher than in previous studies [14]. However, the Cmin obtained for the 100 mg dose in both the deltoids and gluteus (36.1) is more similar to that of the SmPC. As can be seen, there is a wide variability in the Cmin reached between the different studies available in the literature. Despite this variability, the Cmin obtained in our work would be within the therapeutic reference range proposed by the AGNP, but taking into account the values individually, 45.4% of the patients are outside the therapeutic range in Ctrough [5].

With regard to Cmax, Carabias et al. achieved a geometric mean (CV) of 47.4 (38.9) after gluteal administration of 75 mg, which is slightly lower than the value obtained in the present study, 57.35 (33.3) for the same dose and administration site. On the other hand, some authors reported a geometric mean for Cmax (CV) of 57.7 (39.3) for deltoid administration of 75 mg, which was similar to the value obtained in our study, which was 53.48 (27.25) for the same conditions [22].

In the case of Walling et al., the authors reported a geometric mean (CV) Cmax value of 64.85 (39.8) for gluteal administration of 100 mg, which is very similar to the value obtained in the present study for the same dose and administration site, which was 59.05 (28.64) [29].

Based on its SmPC, Cmax plasma concentrations of the active moiety after repeated administration of Risperidone ISM^®^ intramuscular injections of 75 mg and 100 mg are 35.9 and 69.7 ng/mL, respectively [14]. With regard to the administration site, it addresses that the average steady-state exposure was similar for the gluteal and deltoid injection sites. In this sense, in our case for the 100 mg dose, we found a certain difference according to the site of administration, with a 23.9% higher exposure in the deltoid administration compared to the gluteal.

Regarding the area under the curve (AUC) data, the geometric mean (CV) results reported by Carabias et al. were 17,300 (27.1) for a gluteal administration of 75 mg [22], a value lower than that obtained in the present study, which reached 24,295 (43.3). Similarly, the results obtained in the same work were 18,500 (43.3) for deltoid administration of 75 mg, again lower than those observed in the present study for the same dose and administration site, which were 24,561 (62.8). This implies, in comparison, a 40% higher exposure in our study. However, in the study by Walling et al., an AUC for the dosing interval of 25,968 h*ng/mL was obtained for Risperidone ISM**^®^** 100 mg administered in the gluteal region [29], which is similar to that obtained in the present work (23,442).

Although the mean plasma concentrations achieved by risperidone ISM**^®^** in the present study are within this proposed therapeutic range, a high variability among patients can be observed. In order to explain possible causes of this variability, one reason could be the preparation of the formulation by healthcare personnel prior to injection. It is necessary to reconstitute the prefilled syringe of powder with the prefilled syringe of the solvent provided immediately before administration. If the reconstitution process is performed incorrectly, this could affect the proper dissolution of the powder, and if administered, a higher peak of risperidone in the first few hours (overdose) and a lower AUC of the full treatment dose (underdose) may be observed. Another explanation might be differences in the composition of body fluids at the injection site between patients, leading to varying rates of polymer degradation [14].

This study has several limitations, among which the small sample size should be emphasized, as it may prevent certain observed differences from reaching statistical significance. Additionally, the timing of samples collected throughout the dosing period is relatively limited due to the observational nature of the study.

In conclusion, the ISM formulation of Risperidone achieves mean plasma concentrations within the therapeutic range within the first few hours in a considerable group of patients. Additionally, this formulation maintains consistent plasma concentration fluctuations throughout the therapeutic interval. However, there is significant interindividual variability in plasma concentrations. This variability is evident both in previously published studies that supported the drug’s approval and in the present study.

Understanding the causes of such variability, which may include factors such as differences in the injection site, body composition, or preparation and administration techniques, could pave the way for optimized dosing strategies. This, in turn, could lead to improved efficacy and safety of Risperidone ISM therapy, particularly in subgroups of patients who are currently outside the therapeutic range. Moreover, the findings underline the importance of TDM in clinical practice to ensure personalized treatment approaches and better patient outcomes. Additionally, research conducted in real-world clinical practice settings is essential to better understand the impact of these findings on long-term clinical outcomes, including treatment adherence, relapse rates, and adverse effects. Nevertheless, further studies with larger sample sizes should be performed in order to relate the observed variability to individual patient characteristics and strengthen the available scientific evidence regarding these findings.

## Figures and Tables

**Table 1:** Baseline and demographic characteristics. IQ: interquartile range; Cr: Creatinine; AST: Aspartate Aminotransferase; ALT: Alanine Aminotransferase; GGT: Gamma-Glutamyl Transferase; SD: standard deviation.

	Risperidone ISM^®^ 75 mg	Risperidone ISM^®^ 100 mg	Overall
n	10	34	44
Age median (IQ) years	50 (40.7–68.5)		51 (41–62)
Sex [n (%)]			
Male	5 (50)	23 (67.4)	28 (63.6)
Female	5(50)	11 (32.3)	16 (36.3)
Injection site			
Deltoid [n (%)]	3 (30)	10 (29.4)	13 (29.5)
Gluteal [n (%)]	7 (70)	24 (70.5)	31 (70.4)
Height [mean (SD)] (cm)	167.4 (11.5)	169.6 (8.2)	169.1 (9)
Weight [mean (SD)] (Kg)	82.03 (20.9)	86.51 (17.6)	85.49 (18.2)
BMI [mean (SD)] (Kg/m^2^)	29.18 (6.5)	30.3 (6.9)	30.05 (6.8)
Cr (mg/dL)	0.79 (0.18)	0.81 (0.2)	0.81 (0.2)
AST	18.2 (4.3)	20.55 (9.2)	19.83 (8)
ALT	25.21 (9.7)	27.55 (12.6)	26.9 (11.8)
GGT	23.26 (8.2)	28.17 (14.8)	26.67 (13.3)

**Table 2:** Plasma pharmacokinetics parameters for active moiety after the fourth dose of Risperidone ISM^®^.

	Risperidone ISM^®^ 75 mg		Risperidone ISM^®^ 100 mg	
AUC (h*ng/mL) Geometric Mean (CV)	24,428 (49.15)		24,971 (41.2)	
	Deltoid	Gluteal	Deltoid	Gluteal
	24,561 (62.8)	24,295 (43.3)	29,048 (40.9)	23,442 (41.6)
Cmin (ng/mL) Geometric Mean (CV)	34.4 (58.1)		36.1 (45.8)	
	Deltoid	Gluteal	Deltoid	Gluteal
	35.8 (77.4)	33.64 (51.8)	35 (45.7)	36.6 (46.9)
Cmax (ng/mL) Geometric Mean (CV)	55.7 (27.9)		59 (32.5)	
	Deltoid	Gluteal	Deltoid	Gluteal
	53.48 (27.25)	57.35 (33.3)	58.88 (43.53)	59.05 (28.64)
Tmax (h) Median (Min-Max)	24 (2–24)		24 (2–24)	
	Deltoid	Gluteal	Deltoid	Gluteal
	2 (2–24)	24 (24–24)	24 (2–24)	24 (2–24)

## Data Availability

The datasets presented in this article are not readily available because they are part of an ongoing study. Requests to access the datasets should be directed to the corresponding author.
